# Acute stress impairs visual narrative comprehension in younger but not older adults

**DOI:** 10.1038/s41598-026-47338-4

**Published:** 2026-06-12

**Authors:** Ekaterina Varkentin, Irina R. Brich, Kurmanzhan Kurmanbekova, Markus Huff

**Affiliations:** 1 Perception and Action Lab, Leibniz-Institut für Wissensmedien, Schleichstraße 6, 72076 Tübingen, Germany; 2 Department of Psychology, University of Tübingen, Tübingen, Germany

**Keywords:** Narrative comprehension, Age, Acute stress, Age comparison, Psychology, Cognitive ageing

## Abstract

Visual narrative comprehension is essential for navigating modern society, where information, rules, and news are frequently communicated through images, diagrams, and visual stories. Encoding a coherent narrative from disparate elements is critical for all age groups. Although recent studies report a significant rise in stress and anxiety levels, driven by factors such as the COVID-19 pandemic and recent geopolitical conflicts, the impact of stress on visual narrative comprehension remains largely underexplored. This study explored how acute stress affects narrative comprehension in younger (*N* = 203, 18–57 years; *M* = 23 years; Experiment 1) and older adults (*N* = 212, 60–85 years; *M* = 67 years; Experiment 2). Participants were assessed under both acute stress and neutral conditions. A tool for inducing acute stress online employed mathematical and logical tasks under time pressure, along with elements that simulate social stress. Participants were presented with pictorial stories consisting of three panels, with the second panel intentionally left blank. Their task was to comprehend the storyline despite the missing information. On the following page, an image representing a possible bridging event was shown, either depicting the correct or an incorrect inference. Participants were asked to judge whether or not the presented image accurately reflected the missing event in the story. Results revealed that acute stress negatively impacted narrative comprehension in younger adults, while the older adults’ comprehension remained unaffected by acute stress. Similarly, younger adults demonstrated reduced confidence in their responses under stress, whereas older adults’ confidence levels remained unaffected. These findings highlight the relationship between visual narrative comprehension, stress, and aging, suggesting that, with age and experience, comprehenders may develop more differentiated event schemas, which makes their comprehension processes more resilient to stress. Understanding how cognitive and perceptional processes function under stress is crucial for daily life across all age groups. Our research demonstrates that younger adults exhibit poorer visual narrative comprehension under acute stress, whereas older adults’ performance remains stable. This finding suggests that older adults may employ more differentiated event schemas, which help maintain their narrative comprehension in the face of stress. Consequently, narrative comprehension appears to be more resilient compared to other fundamental cognitive skills. These insights could inform interventions and strategies to support cognitive health across different age groups.

## Introduction

### Acute stress impairs visual narrative comprehension in younger but not older participants

Narrative comprehension is crucial for understanding and processing information in the increasingly visual world. This ability, underpinned by cognitive frameworks such as the Structure-Building Framework^1^ and Visual Narrative Grammar^2,3^, is essential across all age groups. With stress becoming an ever-present factor in modern life, understanding its impact on cognitive functions like narrative comprehension is vital, as stress can both impair and facilitate performance^4^. Aging is associated with changes in cognitive processing, including slower information processing, reduced working memory capacity, and differences in emotion regulation^5,6^, which may alter how stress affects narrative comprehension. Older adults may experience stress differently and show distinct susceptibility to its cognitive effects, making it important to examine age-related differences in stress-induced comprehension impairment to clarify how stress interacts with cognitive aging.

### Narrative comprehension

Narratives are important in many aspects of everyday life, as they convey knowledge, information, instructions, and explanations. Understanding narratives is therefore crucial for participating in social life and accessing education and knowledge. Making sense of a narrative is known as *narrative comprehension*^7^, which happens by drawing meaning from incoming information^8^ and generating a mental representation of the narrative’s components^9^. While much is known about how people generally comprehend narratives, far less is understood about how aging and stress interact to influence this process. This represents a critical gap, given that both factors are known to shape cognition in distinct ways. Studying aging in this context is especially important: narrative comprehension draws on multiple cognitive functions (e.g., memory, attention, inferencing, and integration of information), many of which show age-related decline, while others, such as accumulated knowledge, remain stable or even improve. Similarly, stress is known to modulate attention and memory, which are key to successful comprehension. Thus, examining narrative comprehension in aging not only informs us about a vital real-world skill but also provides a window into how age- and stress-related cognitive changes interact in shaping everyday understanding.

Narrative comprehension relies on several core cognitive skills. Working memory supports the temporary maintenance and integration of narrative information, while attention guides the selection of relevant details and the suppression of irrelevant ones^10^. Long-term memory contributes by providing prior knowledge and schemas that support inference-making and global coherence. Inferencing in narrative comprehension is the process of drawing conclusions about unstated events or motivations by integrating existing cues. Together, these mechanisms allow individuals to segment events, track characters, and build mental models that represent unfolding storylines^11,12^.

Visual narratives, which are conveyed through visual codes such as images, comics, or picture sequences, are a particularly widespread medium of communication. They span children’s books, comics, instructional materials, social media, and educational tools. Understanding visual narratives involves not only perceptual processing but also the construction of coherent meaning across sequential images. The structure of visual narratives itself makes inferencing indispensable. Narratives, especially in visual forms such as comics, almost always contain gaps in the representation of events. It is not possible to show action in a completely continuous manner; instead, discontinuities are inherent, and readers must actively fill these gaps. Thus, the generation of inferences is not only supportive but a necessary process for visual narrative comprehension^13–16^.

To conceptualize processes of visual narrative comprehension, several theoretical frameworks have been proposed. For example, the Structure-Building Framework^1^ emphasizes that comprehension involves creating a cohesive mental representation of incoming information by laying a foundation, mapping new information, and updating mental structures through the enhancement of relevant and suppression of irrelevant information. Gernsbacher and Faust^17^ further proposed a general comprehension skill, reflecting the capacity to understand both linguistic and nonlinguistic information. The Scene Perception and Event Comprehension Theory (SPECT) and study by Magliano et al.^18,19^ distinguishes between “front-end” processes, such as attention and information extraction from visuals, and “back-end” processes, such as inference-making, event segmentation, structure building and event-model updating. Finally, Visual Narrative Grammar (VNG)^2,3^ suggests that sequences of images follow a grammar-like structure, comparable to the syntactic organization of sentences. Within this framework, perceptual–semantic updating and narrative-structural processing interact dynamically^2,20^. During visual narrative comprehension, the brain integrates this narrative structure along with more general semantic schemas^21^ to build global narrative coherence and support efficient semantic processing of the images^3,22,23^.

Although these frameworks differ in emphasis, they converge in describing narrative comprehension as the dynamic integration of perceptual input, memory, and inferential reasoning. Importantly, none of these accounts directly address how aging and stress shape comprehension. Thus, after situating our study in relation to these frameworks, we turn to empirical findings on aging and stress that may influence narrative comprehension.

### Narrative comprehension in older adults

Research findings on the impact of age on visual narrative comprehension are ambiguous. There is evidence that perception and attention change with age, which may in turn influence how narratives are understood. For example, older adults tend to segment events in videos differently than younger adults, perceiving event boundaries more distinctly^24^. These differences in attention and event segmentation could affect narrative comprehension by shaping how information is organized and integrated. A more segmented representation might help older adults recall specific details, but it could also change the perceived coherence of the story, suggesting that age-related differences in comprehension may arise from differences in how events are attended to and mentally structured.

Moreover, research indicates variations in certain facets crucial to narrative comprehension, such as age-related discrepancy in character tracking abilities during narrative processing. Older readers face distinct challenges in both identifying the initial character after the introduction of a new character and in comprehensively encoding a new character while multiple characters coexist within the discourse context^25^. Regarding reading comprehension, adults aged 70 and older may face more difficulties than younger adults aged 55 to 69. However, the performance of adults aged 70 and older on multiple-choice inferential questions still meets or exceeds normative control scores, indicating that they retain the basic reading comprehension skills necessary for everyday life^26^. Also, syntactic processing operations remain stable with age^27^. Ulatowska and colleagues^28^ found in a longitudinal study that the ability to generate global inferences remains consistent across different ages. For instance, they assessed 16 healthy older adults in their 80 s and 90 s at two separate testing times and found no significant age differences in forming global representations of text. However, the small sample size limits the generalizability of these findings. Newer studies with larger sample sizes (*N* = 1487, *N* = 142) demonstrate that despite widely known age-related changes in memory and cognitive function, narrative comprehension remains stable in older adults^16,29^. For example, Huff et al. reported this finding in a representative German sample (*N* = 1487), and Varkentin et al. replicated it in a separate sample of older adults (*N* = 142)^16,29^.

### Stress and narrative comprehension

Individuals across the lifespan encounter stress; however, stress perception changes with age, with younger adults typically reporting higher levels of daily and domain-specific stress than older adults^30^. Longitudinal research further indicates that, although exposure to stressors may increase with age, perceived stress often decreases over time, consistent with improved coping and emotion regulation in later adulthood^31,32^. Importantly, acute stress can influence cognitive processes relevant to narrative comprehension, regardless of age, including working memory^33^, attention (including inhibitory control and selection)^34^, and episodic memory^35^. Thus, narrative comprehension is a cognitive process that can be affected by acute stress experiences^36^. During acute stress, a complex response of the human body and brain is activated. While some studies demonstrate negative effects of acute stress on cognitive performance^36–39^, others report the opposite pattern^36,40^, suggesting that acute stress affects different cognitive domains in distinct ways or even showing performance improvements under stress^36,40^.

More specifically, evidence suggests that stress adversely affects working memory^37–40^. For example, Oei et al.^37^ and Xin et al.^38^ found evidence of reduced working memory capacity after acute stress induction. Schoofs et al.^39^ showed that exposure to acute stress leads to the impairment of working memory in a numerical n-back test. However, this impairment weakens with task duration. Working memory is closely linked to inhibitory processes, including the suppression of irrelevant or no-longer-needed information, stored in memory^41^. In the Structure-Building Framework^1^, as described by Gernsbacher et al., suppression is essential for shifting from one mental structure to another when new, unrelated information is encountered, thereby maintaining coherence in comprehension. Similarly, Oakhill et al. emphasize that successful comprehension requires the suppression of misleading or irrelevant interpretations so that the correct inferences can be generated^42^. Acute stress, by impairing working memory, may therefore weaken suppression processes, leading to less efficient construction of a coherent situation model and poorer narrative comprehension.

Behavioral and neuroimaging studies have reported stress-related impairments in inhibitory control, including response inhibition and conflict monitoring, even in cases where behavioral performance remains unaffected^36^. Other studies have observed preserved or even enhanced inhibitory performance under stress, suggesting that the impact of stress on inhibition may depend on task demands, timing, and the level of control required. Together, these findings indicate that stress can directly affect inhibitory mechanisms, particularly those relying on controlled, prefrontal processes.

Importantly, studies directly examining the effects of stress on inhibition indicate a complex and nuanced relationship^36^. At the neural level, acute stress appears to reallocate cognitive resources, potentially enhancing early, automatic inhibitory processes while impairing later, more controlled forms of inhibition^43^. Behavioral and neuroimaging studies have reported stress-related impairments in inhibitory control^44^, including response inhibition and conflict monitoring, even in cases where behavioral performance remains unaffected^45^. Other studies have observed preserved or even enhanced inhibitory performance under stress^46–48^, suggesting that the impact of stress on inhibition may depend on task demands, timing, and the level of control required. Together, these findings indicate that stress can directly affect inhibitory mechanisms.

Given that aging is commonly associated with declines in inhibitory control^49,50^, older adults may already experience reduced efficiency in suppressing irrelevant information during comprehension. Acute stress may therefore exacerbate these age-related inhibitory limitations, either directly or indirectly via stress-related working memory impairments, potentially amplifying difficulties in constructing and maintaining coherent narrative representations.

Attention, which encompasses selection and inhibitory control, is another important cognitive process for narrative comprehension and inference generation. Domes and Zimmer^51^ found in their study that participants exposed to the Trier Social Stress Test^52^ showed an improved identification of emotional cues. It is assumed that this might help detect any threats and also act as a support system in social situations^51^. Shields et al.^40^ revealed that exposure to mild acute stressors was associated with increased response speed in two attentional tests without affecting response accuracy. Other authors show similar results, with acute stress enhancing attention^53,54^. To the best of our knowledge, no research papers investigate the association between acute stress and narrative comprehension or inference generation. Therefore, we intend to investigate whether exposure to acute stress may weaken participants’ ability to comprehend narratives and draw correct inferences.

### Confidence in narrative comprehension

An important but often overlooked aspect of narrative comprehension is not only whether individuals arrive at correct interpretations, but also how confident they are in their own understanding. Confidence judgments reflect metacognitive monitoring, which is the ability to evaluate one’s own performance. It plays a crucial role in regulating learning and memory processes^55^. Prior work shows that stress can reduce the sensitivity and reliability of confidence judgments (metacognitive monitoring), meaning that individuals become less aligned between confidence and actual performance^55^. In a study on visual perception and confidence, confidence efficiency was reduced in older adults, meaning that while they can still judge their performance, their confidence judgments are less efficiently aligned with actual performance, often due to declines in cognitive control^56^. Although confidence has not been systematically examined in the context of visual narrative comprehension, it is plausible that both stress and age-related changes in metacognitive control affect how individuals judge their own narrative understanding. To address this, we included an exploratory analysis of confidence in participants’ responses in addition to examining comprehension accuracy.

### Experimental overview and hypotheses

The present study tested the influence of experimentally induced acute stress, as reflected in participants’ perceived stress levels, on visual narrative comprehension in a younger student sample (*N* = 203 after exclusions, 18–57 years; *M* = 23 years; Experiment 1) and in an older sample (*N* = 212 after exclusions, 60–85 years; *M* = 67 years; Experiment 2). The younger sample was recruited through a university mailing list restricted to currently enrolled students. As expected, most participants fell within the 18- to 30-year-old age range. However, due to occasional exceptions in university enrollment, a few participants were slightly above that age range. To ensure that these exceptions did not influence our findings, we conducted an outlier analysis, which is reported in section Robustness checks in the Results. Considering the lack of prior evidence about the effect of acute stress on narrative comprehension, we stated a non-directional hypothesis and expected narrative comprehension to differ between the stress condition and the control condition in Experiment 1 (younger sample) (H1; preregistered here: https://aspredicted.org/Y11_CM2). Anticipating the results of Experiment 1, which showed that acute stress reduced narrative comprehension in younger adults, we formulated a directional hypothesis in Experiment 2 and expected acute stress to negatively affect narrative comprehension in older adults (H2; preregistered here: https://aspredicted.org/7C2_VBF). In addition, we tested the following exploratory hypotheses in each experiment: firstly, we explored whether there is a difference in confidence responses between the stress and control conditions (Participants were presented with the question, “How confident are you in your answer?” with six response options ranging from “very uncertain” to “very confident”). Secondly, we investigated whether narrative comprehension decreases with an increased degree of difficulty.

The experiments have a between-subjects design. The experiments manipulated stress induction as an independent variable (between-subjects) with stress-invoking stimuli in the experimental (stress) condition and easy filler tasks in the control condition. We measured narrative comprehension by testing bridging inference generation (i.e., inferences generated to bridge gaps in short pictorial stories)^13,16,29,57^. Linking consecutive events within a story is essential for comprehension. That is why, by measuring this process, we can draw conclusions about overall narrative comprehension skills. This method is an established experimental procedure to research inference generation and, thus, narrative comprehension of both textual and pictorial stories. Similar to Huff et al., Magliano et al. and Varkentin et al.^13,16,29,57^, we used a bridging event generation measurement in our study. Bridging events in comics/picture stories are panels depicting information that connects the narrative gap between two elements (panels). In our study, the bridging event of the stories was not presented to the participants and had to be inferred for comprehension after viewing only the stories’ beginning and end state. Participants saw a blank white panel instead of the bridging event. If a bridging event is missing in a visual or textual story, it must be inferred from the information presented in the beginning and end states. This process consumes cognitive resources that can be measured in different ways, for example, by answering comprehension questions^1,16,29^. Our study applies this method and tests the formation of inferences between the beginning and end state. After each story, participants receive one picture either depicting the correct inference for the missing bridging event or a false inference to be judged for correctness. Confidence in this answer was also assessed in order to evaluate metacognitive awareness.

### Overview of measures and analyses

Across both experiments, acute stress induction (stress vs. control) served as the primary between-subjects independent variable. Narrative comprehension, operationalized via accuracy in a visual bridging-inference task, was the primary dependent measure. Subjective stress and affect were assessed both before (Timepoint 1) and after (Timepoint 2) the stress induction or control task to validate the effectiveness of the manipulation. In addition to the preregistered hypotheses for each experiment, we conducted an exploratory analysis examining the potential interaction between age group (younger vs. older adults) and stress condition on narrative comprehension. This analysis was intended to assess whether the effect of acute stress on comprehension differs between younger and older adults, providing insight into possible age-related moderation of stress effects.

In addition to the preregistered hypotheses testing stress-related differences in narrative comprehension (Experiments 1 and 2), exploratory analyses examined confidence judgments as a measure of metacognitive awareness and the effect of story difficulty on comprehension performance. Table 1 provides an overview of the experimental measures, variables, and analyses conducted across both experiments.

**Table 1:** Overview of experimental variables, measures, and analyses.

Category	Variable/measure	Purpose
Independent variable	Stress condition (stress vs. control)	Experimental manipulation
Manipulation check	Subjective stress (VAS), PANAS (pre/post)	Verification of stress induction
Covariates/descriptive	Perceived Stress Scale (PSS), Stress Coping Inventory	Characterization of sample
Primary dependent variable	Narrative comprehension (bridging inference accuracy)	Test of H1 and H2
Exploratory dependent variable	Confidence ratings	Metacognitive awareness
Task characteristic	Story difficulty level	Exploratory analysis
Between-subjects factor	Age group (younger vs. older adults)	Cross-experiment comparison, exploratory analysis examining the potential interaction between age group (younger vs. older adults) and stress condition on narrative comprehension.

## Methods

### Transparency and openness

#### Transparency in data, analysis, and materials

We affirm that the de-identified data on which the study conclusions are based are available at the project’s Open Science Framework page (https://osf.io/5q3xc/overview).

We affirm that the syntaxes used to conduct analyses in the statistical package R are available at the project’s Open Science Framework page (https://osf.io/5q3xc/overview). For data analysis we used R (version 4.3.1) and RStudio (version 2023.12.0).

**Fig. 1:**
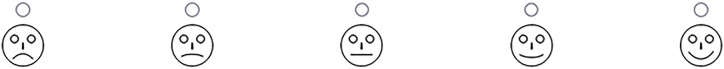
Assessment of the perceived story’s affect in the pretest.

**Fig. 2:**
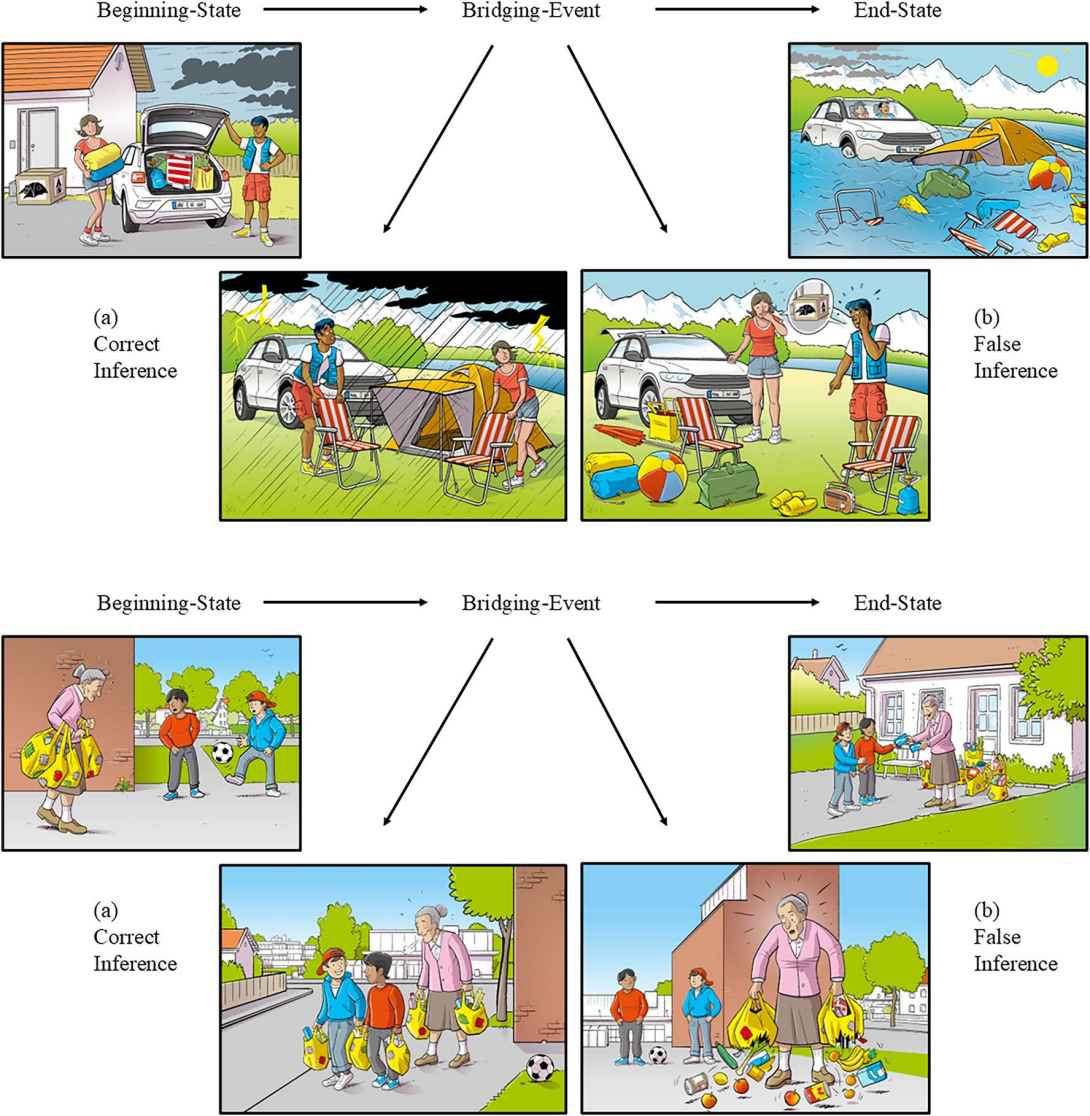
Example of a story from Level 2 (upper story) and 3 (lower story). The upper row shows the beginning state and end state, which were presented to the participants with a blank panel between them instead of the bridging event. The lower row shows two possible inferences for the bridging event. All drawings were commissioned from professional illustrator Reinhart & Schlegel GbR; copyright was transferred to the authors, who release the figure under the Creative Commons Attribution 4.0 International license (CC BY 4.0).

We present an example of the study materials (see Figs. 1, 2). All visual materials employed in this study can be retrieved by contacting corresponding author (e.varkentin@iwm-tuebingen.de). Access to these materials requires prior acceptance of the corresponding copyright and license agreement, as they were developed by a team of researchers in the Leibniz Institut für Wissensmedien (IWM).

#### Transparency in research design

We affirm that we report how we determined our sample size, all data exclusions, all manipulations, and all measures in the upcoming sections Participants. If specific exclusions were еrequired due to missing data in particular values, we have documented such occurrences in the section Results.

#### Preregistration

Both parts of the study were preregistered (including study design, hypotheses, and analytic plan): https://aspredicted.org/Y11_CM2; https://aspredicted.org/7C2_VBF.

#### Material test

We conducted a stimulus norming procedure (referred to here as the Material Test) to evaluate difficulty and affect for each story and select the final set of 24 stories. As no standardized materials had been developed yet to measure narrative comprehension, and the existing materials were primarily oriented toward child samples, we first developed a set of textual and pictorial stories to measure narrative comprehension. Initially, textual materials were developed, and we conducted a pretest of the textual stimulus material in a student sample. A total of 28 items were developed, with three difficulty levels in the text form. Difficulty levels were differentiated based on the number of storylines within a story. At level 1 (simplest), there was one storyline, and, in most cases, stories involved a single character. At level 2, there were two storylines and more than one character. At level 3, there were at least three storylines and even more characters (see Fig. 1). Moreover, with increasing difficulty, we had more details in each story, which is why each level had a different number of words, i.e., level 1 had the shortest stories (30–40 words per panel), while levels 2 (40–50 words per panel) and 3 (40–60 words per panel) were consequently longer. Additionally, we made sure to develop both positive (e.g., boys helping an older woman) and negative scenarios (e.g., flooded camp site) to counterbalance the sentiment of a story. The text items were evaluated in a preliminary study with 28 participants. To assess perceived affect, participants were asked, *“How would you describe the mood of the story?”* with five response options presented as face icons representing varying emotional expressions (see Fig. 1).

Next, participants were prompted to describe what they believed happened between the two parts of the story (i.e., in the empty frame). This allowed us to evaluate whether the bridging inferences generated by the research team aligned with those spontaneously produced by readers.

To assess perceived difficulty, participants answered the question, *“How difficult was it for you to understand the plot of the story?”* on a 5-point-Likert scale with the following options: *easy*,* fairly easy*,* moderate*,* fairly difficult*,* difficult*.

We also tested the effectiveness of the false inference manipulation. Next, participants were shown the false inference and asked, *“How certain are you that the following action did not occur in the empty frame?”* Responses were recorded on a 5-point-Likert scale: *uncertain*,* somewhat uncertain*,* undecided*,* somewhat certain*,* certain*.

To assess text clarity, participants were asked, *“How challenging did you find the text of this story?”* with the five response options: *easy*,* somewhat easy*,* medium*,* somewhat challenging*,* challenging*.

Finally, an open comment field was provided for participants to share any additional feedback or remarks about each story.

We fitted a linear mixed model with the help of the lme4 package^58^. Results showed that, on average, the subjects rated the items’ affect as we had planned (*OR* = 2.58, *CI* = 2.32–2.84, *t*(25.48) = 19.50, *p* <.05). In terms of difficulty, there were significant differences between the levels 1 and 2, *t*(22.34) = 2.25, *p* =.035, as well as between the levels 1 and 3, *t*(23.53) = 2.58, *p* =.017. Responses to the open-ended question were evaluated manually via content and meaning analysis. Based on these results, four items that did not yield clear outcomes were excluded. The final set of text items included 24 stories.

The pictorial version was created based on the pretested text stories. To create a pictorial version of items, we worked in close collaboration with a comic artist, who created 24 comics for each textual story. The most crucial issue was that the comics were as equivalent to the text version as possible to make measurements in two codalities (pictorial and textual) in future studies possible. Thus, the development of each story involved multiple feedback loops. Two of the stories (in pictorial) are depicted in Fig. 2. In the current study, we used only the pictorial version as we are interested in measuring visual narrative comprehension.

#### Samples

In Experiment 1, we tested 232 participants (*N* = 203 after exclusions, *M* = 23.26 years, range 18–57 years, *SD* = 5.67, Md = 22 years, 156 female, 44 male, 3 diverse). A total of 101 participants took part in the stress condition, and 102 participants in the control condition. Participants were recruited via email from the University of Tübingen and the University of Trier. The participants participated in exchange for course credit, or they had a chance to win one of two vouchers from a local bookstore.

In Experiment 2, we recruited a sample of 236 participants over 60 years old (*N* = 212 after exclusions, *M* = 67.32 years, range 60–85 years, *SD* = 5.47, Md = 67 years, 77 female, 135 male) with the online-panel provider bilendi & respondi (bilendi.de), which compensated participants for their participation. A total of 90 participants took part in the stress condition, and 122 participants in the control condition. We are unable to provide the racial distribution of the sample for both studies, as questions related to ethnical background are not included in the studies conducted in Germany due to legal restrictions.

In *Experiment 1* (younger sample), the following exclusion criteria were applied: participants with a completion time of less than 10 min or more than 60 min were excluded. However, no participants met this criterion. 24 participants were excluded for having previously participated in an experiment using the same stimuli. Additionally, 2 participants were excluded for not providing consent, and 3 participants requested to withdraw their data. Age-related exclusion criteria were also applied, excluding participants under 18 and over 60 years old, though no participants met this criterion.

In *Experiment 2* (older sample), 22 participants were excluded for not providing consent, and 2 participants requested to withdraw their data. Another exclusion criterion was applied to participants who reported they could not properly see more than half of the presented stories. However, no participants met this criterion.

Both studies received ethical approval by the local ethics commission of the Leibniz Institut für Wissensmedien (IWM) (LEK 2022/043). All methods were performed in accordance with the relevant guidelines and regulations.

#### Procedure

Both experiments were nearly identical, with only two adjustments made in Experiment 2 (older sample): the level of induced stress and the presentation time of the story clips. These changes were based on feedback from the local ethics committee. Importantly, the manipulation remained effective, as confirmed by a successful manipulation check (see Results section). Specifically, we observed significant differences in stress levels between timepoint 1 and timepoint 2, indicating that the stress induction was effective in both the younger and older stress groups.

**Fig. 3:**
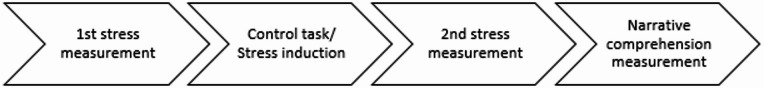
Overview of the procedure.

The experiment was programmed and hosted with SoSciSurvey (soscisurvey.de) as an online study, and participants were randomly assigned either to the stress or control condition. The experiment was conducted in German and began with providing general information about the study goals. However, participants were initially not told that stress induction was part of the experimental design. Instead, they were only told that mathematical and cognitive tasks would be presented. All participants provided informed consent prior to enrollment in the study. Afterward, demographic data were collected, including age, gender, education, and stress-coping strategies (based on the Stress Coping Inventar^59^). Performance on the Stress Coping Inventory^45^ was analyzed, but no significant associations were found. An overview of the experimental design is presented in Fig. 3.

#### Stress induction

In the stress and control conditions, an affect baseline was established using the Positive and Negative Affect Schedule^60^ (PANAS) and acute subjective stress was measured using a visual analog scale ranging from 1 to 100. In addition, participants completed the Perceived Stress Scale^61^ (PSS) to assess chronic stress.

Participants in the stress condition completed an established online stress-induction protocol (www.stress-plus.herokuapp.com)^62^ consisting of time-pressured cognitive tasks combined with negative performance feedback (e.g., “unfortunately, your performance in this task was not sufficient. Please try harder.”) and unfavorable social comparison cues. Specifically, the stress induction included a math task, a numerical Stroop task, and a mental rotation task, each lasting 110 s, resulting in a total induction duration of approximately six minutes, consistent with prior research using comparable stressors^63^. Tasks were adaptive to always remain demanding yet solvable, encouraging sustained engagement while inducing performance pressure.

To account for ethical requrements and age-related differences in processing speed, task difficulty was set to *hard* for younger adults and *medium* for older adults, and response time limits were adjusted. For all tasks, the main adjustment for older participants was a slightly slower pace (1–3 s longer per response) to accommodate age-related reductions in processing speed and motor control. It is important to note that all tasks were adaptive for all age groups. Older participants initially received slightly simpler items (medium level), such as a math problem requiring two operations (e.g., a multiplication followed by addition), whereas younger participants started with items (hard level) requiring three operations (e.g., two multiplications followed by an addition). Once older participants completed three items correctly, the task difficulty automatically increased to match the level experienced by younger participants. These adjustments ensured that the stress manipulation remained subjectively challenging but not overwhelming for older participants.

Participants in the control condition completed low-demand filler tasks (e.g., semantic categorization such as fruits vs. vegetables) that were matched in duration but did not involve time pressure or evaluative feedback.

Following the stress induction or control task, affect and subjective stress were assessed again in both conditions. Immediately afterward, participants completed the narrative comprehension task described in the subsequent section.

#### Narrative comprehension task

In this task, participants were instructed to comprehend the pictorial stories with a missing middle part (i.e., the bridging event). They could see only the beginning- and end-states of each of the 24 stories. Before the main experiment, we presented two exercise stimuli, demonstrating the experimental manipulations. For presentation, the pictures were shown simultaneously in a row while the middle picture (bridging event) of each story was replaced by an empty frame (see Fig. 2, upper row).

**Fig. 4:**
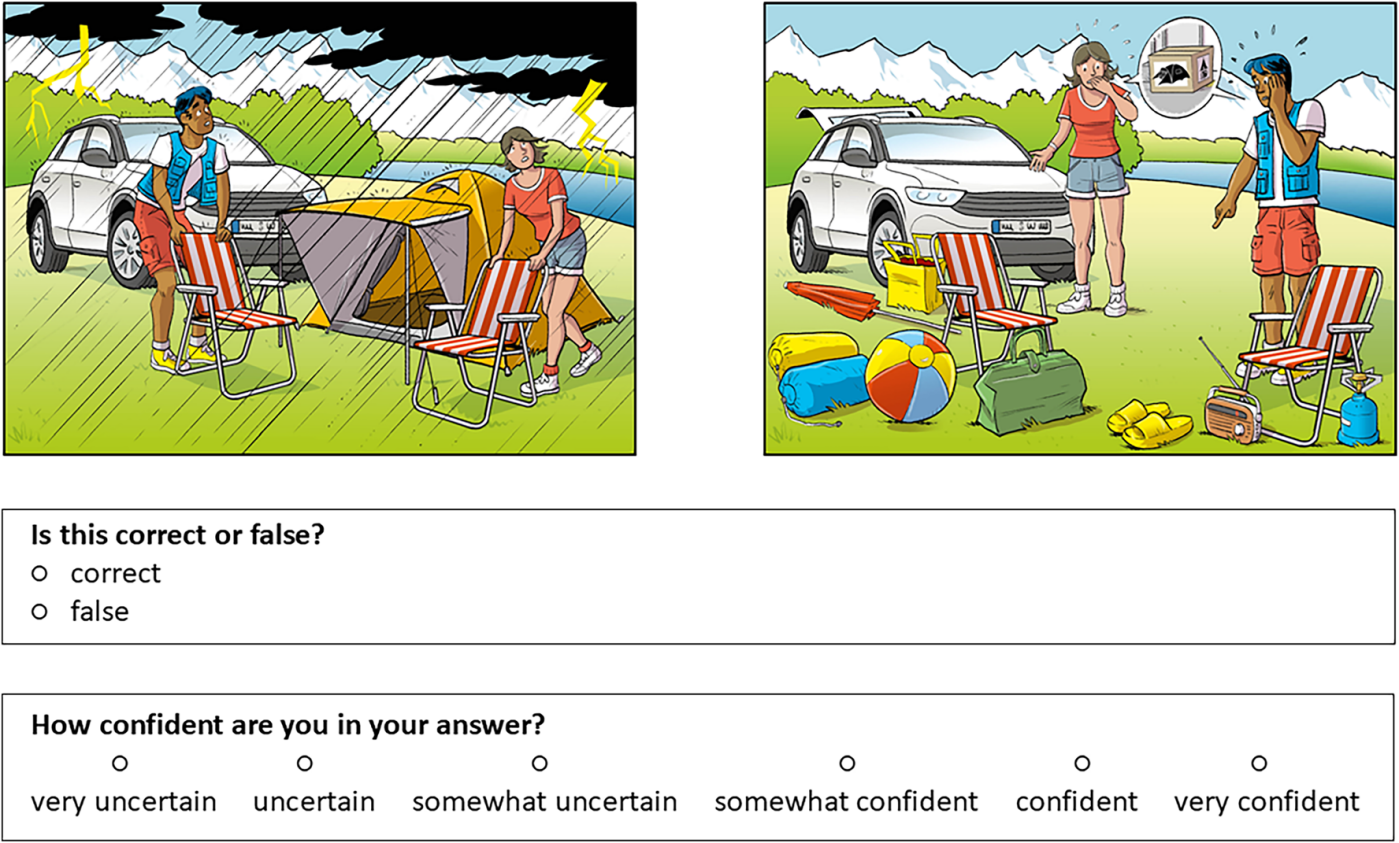
Examples of inferences and questions following the inference recognition task. The left picture shows the correct inference and the right picture shows the false inference for the story depicted in Fig. 2 (upper row). For each story, only one of the pictures was shown in the actual task. The lower row represents the questions in the presentation mode exactly as they were seen by the participants. All drawings were commissioned from professional illustrator Reinhart & Schlegel GbR; copyright was transferred to the authors, who release the figure under the Creative Commons Attribution 4.0 International license (CC BY 4.0).

We set the viewing time of each story to nine seconds for the younger sample and to eleven seconds for the older sample. Upon the time expiry, participants were automatically forwarded to the next page. Here, they received either a correct or a false inference presented in the form of a picture and were asked to indicate whether it matched the story they had been shown. Moreover, the confidence in this answer was also assessed. Participants were presented with the question, “How confident are you in your answer?” with six response options ranging from “very uncertain” to “very confident.” Fig. 4 illustrates both inference options (false on the left and true on the right) for the story presented in Fig. 2, upper row, and portrays the questions in the presentation mode, replicating the participants’ perspectives.

The correct inference contains the bridging event, that is, the plot that connects the beginning- and end-states, while the false inference includes the same characters and same surroundings, but presents a different plot that does not connect the beginning and end states, instead depicting an alternative storyline not matching the ending of the story. Each clip was paired with either a true or a false inference picture regarding the blank frame. For each of the 24 experimental clips, we presented either a true (12 trials) or a false (12 trials) inference. The presentation order of the 24 clips was randomized and the combination with true/false inferences was counterbalanced across participants^16,29^.

After finishing the narrative comprehension task for the 24 stories, participants in Experiment 2 (older sample) were asked whether they could sufficiently see most of the stories in the given time. At the end of Experiments 1 and 2, all study participants were debriefed regarding the study aims. Thus, we could reduce their distress level, which should have been induced in the experimental group.

## Results

First, we will describe the results of Experiment 1 conducted with the younger adults. Second, we will present the findings from Experiment 2, conducted with the older adults. Finally, we will statistically compare the two datasets. Exploratory, we will describe the results of the analysis of the relationship between the narrative comprehension and the story´s difficulty level.

### Experiment 1 (younger sample)

#### Manipulation check

To validate the results of the stress induction, PANAS, and subjective stress measurement took place twice during the experiment in both conditions (control, stress), before and immediately after the stress induction in the experimental condition, and filler matching tasks in the control condition (see Table 2).

**Table 2:** Stress assessment for the younger sample as a function of condition (stress, control) and measurement time (t1: before induction, t1: after induction).

Condition	Scale	t1		t2	
		*M*	*SD*	*M*	*SD*
Stress	PANAS (positive affect) PANAS (negative affect)	26.54 14.88	6.52 5.96	22.74 18.93	6.73 8.09
	Subjective stress	37.19	27.57	54.44	28.48
Control	PANAS (positive affect) PANAS (negative affect)	26.27 13.99	6.50 4.22	24.38 13.66	7.37 4.44
	Subjective stress	37.89	24.84	34.72	23.72

Note. t1: before stress induction or filler matching task, t2: after stress induction or filler matching task.

A t-test for dependent samples revealed a significant increase in the negative affect in the stress condition, *t*(100) = − 6.11, *p* <.001, *d* = 0.61. Those participants also showed a significant decrease in their positive affect, *t*(100) = 7.68, *p* <.001, *d* = 0.76. The participants in the control condition showed no significant differences in the negative affect scale, *t*(101) = 0.99, *p* =.322, *d* = 0.10. However, there was a significant decrease in the positive affect scale in the control condition, *t*(101) = 4.58, *p* <.001, *d* = 0.45. The participants in the stress condition assessed their subjective stress level as significantly higher after the induced stress, *t*(100) = −6.53, *p* <.001, *d* = 0.65. Participants in the control condition showed no significant difference in their subjective stress level, *t*(101) = 2.04, *p* =.978, *d* = 0.20. Furthermore, we compared with the t-test for independent samples the negative affect between both conditions and found that participants in the stress condition had a significantly higher negative affect after the stress induction than participants in the control condition after solving the alternative filler tasks, *t*(154.9) = −5.74, *p* <.001, *d* = −0.80. Further, participants in the control condition showed a significantly higher positive affect than participants in the experimental group with *t*(199.67) = 1.66, *p* =.049, *d* = 0.23. The subjective stress level in the stress condition was found to be higher than in the control condition at the timepoint two (after the stress manipulation or filler task), *t*(193.94) = −5.36, *p* <.001, *d* = −0.38.

#### Statistical analysis

The statistical approach was the same for both experiments, so we describe it here once. For each pre-registered hypothesis, we compared stress and control conditions using a two-step procedure. First, we tested for equality of variances between groups with an *F*-test (var.test in R). When the *F*-test indicated no significant difference in variances, we used a standard independent-samples *t*-test; when variances differed significantly, we used Welch’s *t*-test, which does not assume equal variances.

To complement these frequentist tests, we also conducted Bayesian *t*-tests with a non-informative Jeffreys’ prior, allowing us to directly quantify evidence for both the null and alternative hypotheses. We followed Jeffreys’ (1961) heuristic: Bayes Factors (BF) between 1 and 3 indicate anecdotal, 3–10 substantial, 10–30 strong, 30–100 very strong, and BFs > 100 extreme evidence for the alternative hypothesis. Fractions (1/3, 1/10, etc.) indicate evidence strength for the null hypothesis. Detailed results for each analysis are presented in the sections below.

A similar approach – frequentist *t*-tests supplemented with Bayesian analysis – was used for the exploratory analyses of confidence ratings.

Finally, to compare the two experiments and test for differences between age groups, we conducted a cross-experimental analysis using generalized linear mixed models (GLMMs). This model included condition (stress vs. control) and age group (younger vs. older sample) as fixed effects (main effects and their interaction), and participants and items as random effects. We submitted the resulting model to a Type 2 Anova using the Anova function from the car package^64^.

#### Pre-registered analysis

**Fig. 5:**
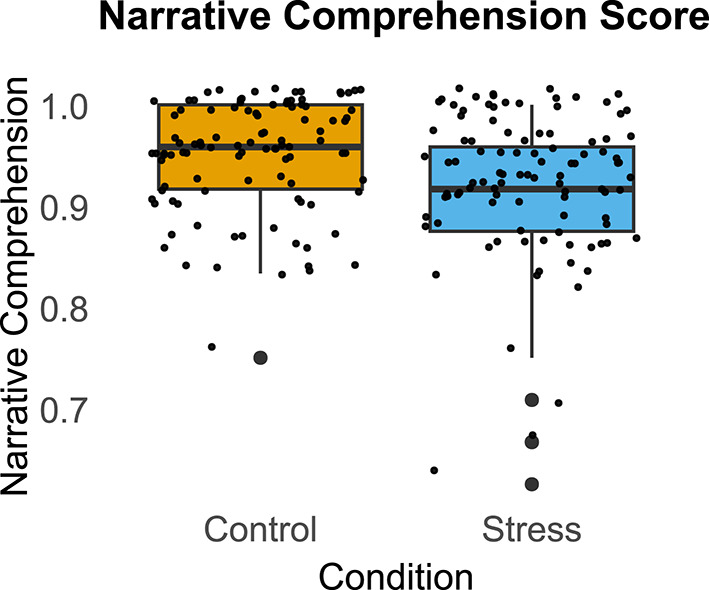
Boxplot depicting the distribution of narrative comprehension as a function of condition (control, stress) for the younger sample.

We tested H1, which predicted the group difference in the narrative comprehension task performance (younger adults in the stress condition would show lower narrative comprehension than those in the control condition). We first calculated the proportion of correct inferences for each participant. We then tested for variance homogeneity. An *F*-test revealed a significant difference between variances, *F*(101, 100) = 0.59, *p* =.008. Accordingly, we used a Welch two-sample *t*-test (frequentist approach), which showed a significant difference between the experimental (stress) and control (no-stress) conditions, *t*(187) = 2.96, *p* =.004, *d* = 0.22. Compared to the control condition, *M* = 0.95 (*SD* = 0.05), narrative comprehension was significantly impaired in the stress condition, *M* = 0.92 (*SD* = 0.07) (see Fig. 5). To account for variability across participants and items, we additionally fitted a linear mixed-effects model with random intercepts for participants and stories; this analysis revealed significant difference between the experimental (stress) and control (no-stress) conditions, χ²(1) =  9.13 *p =* .003.

#### Robustness checks

Because recruitment for the younger group was conducted via a university mailing list, a small number of participants fell outside the typical student age range. To assess whether this influenced the results, the main analysis of narrative comprehension was re-run after excluding the participants who were older than 40 years old. After excluding participants older than 40 years and applying the same general exclusion criteria, the group difference in narrative comprehension remained statistically significant, *t*(180.19) = 3.35, *p* <.001, 95% CI [0.01, 0.05]. Participants in the control condition showed higher comprehension (*M* = 0.95) than participants in the stress condition (*M* = 0.93).

Additionally, we performed an outlier analysis using the 1.5×IQR criterion on individual mean comprehension scores. After excluding these outliers, the group difference remained statistically significant, *t*(195.51) = 2.53, *p* =.012, *d* = 0.36, indicating that the effect is robust and not dependent on a few low-performing participants.

#### Bayesian analysis

To complement the frequentist analysis, we conducted a Bayesian *t*-test with a non-informative Jeffreys’ prior, allowing explicit evaluation of the null hypothesis. The Bayes *t*-test yielded a *BF* = 8.74 which according to Jeffreys’ scale constitutes substantial evidence in favor of the alternative hypothesis – that is, the data are about 8 times more likely under the assumption of a difference between stress and control conditions than under the assumption of no difference.

### Exploratory analyses

#### Confidence transformation

**Fig. 6:**
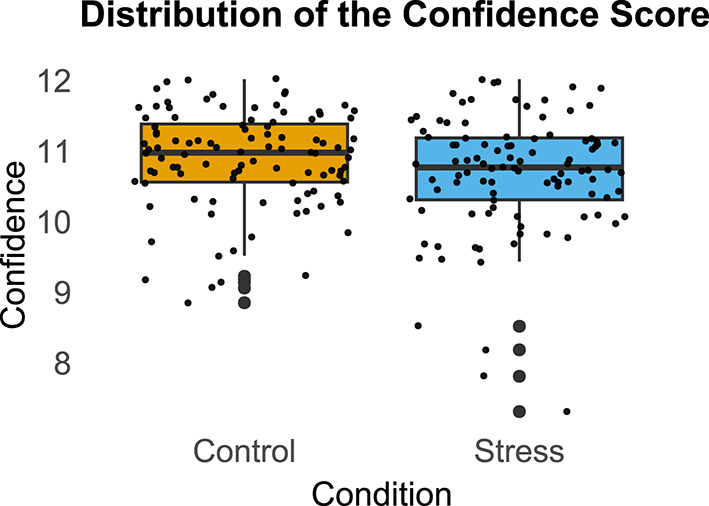
Boxplot describing the distribution of confidence in response in both conditions (control, stress) for the younger sample.

To capture participants’ confidence while taking response accuracy into account, we transformed the original 6-level confidence ratings into a 12-level scale. Each confidence rating was coded separately depending on whether the participant’s response was correct or incorrect, such that lower values corresponded to highly confident incorrect responses and higher values reflected increasingly confident correct responses. This transformation allows the resulting score to reflect *confidence conditional on accuracy*, providing a more informative measure of metacognitive performance than raw confidence alone. In Fig. 6, the 12 levels of the transformed scale correspond to the six original confidence levels split across correct and incorrect responses.

We first explored whether there is a between-group difference in participants’ confidence in their responses. To account for both response correctness and subjective confidence, we transformed each confidence rating into a combined metric ranging from 1 to 12: incorrect answers were mapped to values between 1 (very confident) and 6 (very unconfident), and correct answers to values between 7 (very unconfident) and 12 (very confident). The average of these transformed scores was used as the overall confidence measure for each participant. Participants in the control condition were more confident than participants in the stress condition (*M_control* = 10.87, *SD* = 0.69; *M_stress* = 10.64, *SD* = 0.84), as shown by a t-test for independent samples, *t*(201) = 2.06, *p* =.041, *d* = 0.41 (see Fig. 6).

We additionally calculated a Bayesian *t*-test with a non-informative Jeffreys’ prior. This analysis provided only anecdotal (practically indecisive) evidence for this effect, *BF* = 1.10, meaning the data are nearly equally likely under both the null and alternative hypotheses, and thus do not clearly support one over the other.

### Experiment 2 (older sample)

#### Manipulation check

To validate the results of the stress induction, PANAS and subjective stress measurement took place twice during the experiment in both conditions (stress and control): once before and once immediately after the stress induction in the stress condition and alternative filler tasks in the control condition (see Table 3).

**Table 3:** Stress assessment for the older sample as a function of condition (stress, control) and measurement time (t1: before induction, t1: after induction).

Condition	Scale	t1		t2	
		M	SD	M	SD
*Stress*	PANAS (positive affect) PANAS (negative affect)	30.44 12.51	8.04 4.73	27.53 16.41	7.5 7.15
	Subjective stress	13.26	18.49	37.23	29.17
*Control*	PANAS (positive affect) PANAS (negative affect)	29.70 12.94	7.55 5.2	29.69 12.22	8.01 4.59
	Subjective stress	11.71	18.14	12.39	16.82

Note. t1: before stress induction or alternative task, t2: after stress induction or alternative task.

A t-test for dependent samples was conducted to run the manipulation check. Firstly, we compared the negative affect scores at time point 1 and time point 2 in the stress condition. A t-test revealed a significant increase in the negative affect in the stress condition, *t*(89) = −5.58, *p* <.001, *d* = 0.46. There was also a significant decrease in positive affect in the stress condition, *t*(89) = 5.67, *p* <.001, *d* = 0.59. A paired samples t-test was conducted to compare negative affect scores at timepoint 1 and timepoint 2 in the control condition. There was no significant difference in the negative affect scale, *t*(121) = 1.92, *p* =.057, *d* = 0.17. These results suggest that there was no significant change in negative affect over time in the control condition. The control condition showed also no significant differences in the positive affect, *t*(121) = 0.018, *p* =.986, *d* = 0.00.

The participants in the stress condition assessed their subjective stress level as significantly higher after the induced stress, *t*(89) = −8.62, *p* <.001, *d* = 0.91. This indicates a substantial difference between the two measurements in the stress condition. The participants in the control condition showed no significant difference in their subjective stress level *t*(121) = −0.77, *p* =.222, *d* = 0.07. Furthermore, a t-test for independent samples was conducted to compare the negative affect between both conditions and found that participant in the stress condition had a significantly higher negative affect after the stress induction than the participants in the control condition after solving alternative filler tasks, *t*(141.6) = −4.87, *p* <.001, *d* = 0.68. This suggests that there is a substantial difference in negative affect at timepoint two between the control and stress conditions, with the stress group exhibiting higher negative affect. The control condition showed a significantly higher positive affect than the stress condition, *t*(198.5) = 2.01, *p* =.046, *d* = 0.40. This indicates that there is a moderate difference in positive affect at timepoint two between the control and stress conditions. The subjective stress level in the stress condition was higher than in the control condition at timepoint two (after the stress manipulation or filler task), *t*(132.18) = −7.24, *p* <.001, *d* = 1.09.

#### Pre-registered analysis

**Fig. 7:**
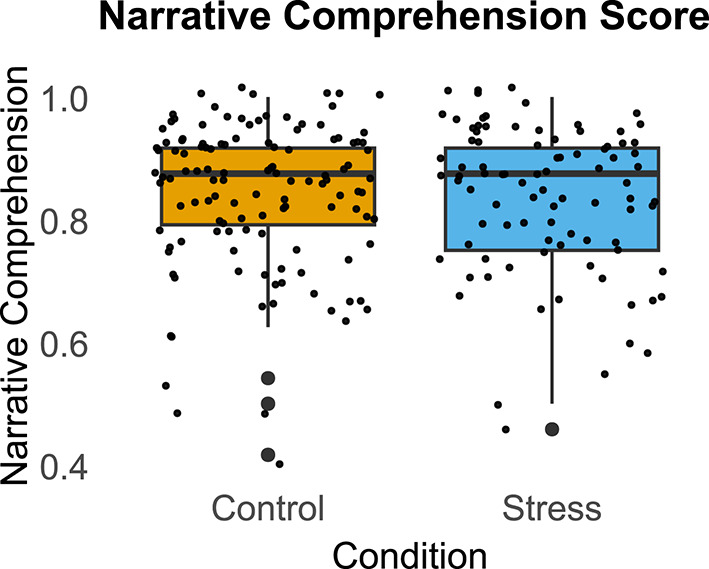
Boxplot describing the distribution of narrative comprehension test performance in both conditions for the older sample.

We tested H2, which predicted that older adults in the stress condition would show lower narrative comprehension than those in the control condition. First, we assessed variance homogeneity using an F-test. Variances did not differ significantly, *F*(121, 89) = 0.94, *p* =.751. We therefore applied a standard independent-samples *t*-test, which showed no significant difference between conditions, *t*(210) = 0.25, *p* =.805, *d* = 0.03. Both groups achieved the same mean score, *M* = 0.84, *SD* = 0.12 (see Fig. 7). A generalized linear mixed-effects model showed no significant effect of stress condition in older adults, χ²(1) = 0.05, *p =*.83, indicating comparable performance across control and experimental groups.

To complement the frequentist analysis, we conducted a Bayesian *t*-test with a non-informative Jeffreys’ prior, allowing explicit evaluation of the null hypothesis. The Bayes *t*-test yielded a *BF* = 0.16, which constitutes substantial evidence in favor of the null hypothesis – meaning the data are about 6 times more likely under the assumption of no difference between stress and control conditions than under the assumption of a difference.

#### Exploratory analyses

The same approach to transforming the confidence variable that was used in the younger sample dataset was applied to the older sample dataset. Further exploratory analysis investigated whether there is a between-group difference in participants’ confidence in their responses and showed that there is no effect of stress on confidence in the older sample. Stress did not affect confidence rating for the correctness and falseness of depicted inferences, (*M_control* =  6.65 *SD* = 1.17 *M_stress* =  6.76 *SD* = 1.12). The conducted t-test for independent samples revealed no significant between-group difference, *t*(210) = −0.708, *p* =.479, *d* = −0.10.

We additionally calculated a Bayesian *t*-test with a non-informative Jeffreys’ prior. This analysis assumes substantial evidence for the null hypothesis, *BF* = 0.25, indicating the data are about 4 times more likely if there is no difference between experimental conditions than if there is a difference.

#### Cross-experimental analysis: comparison of two samples

**Fig. 8:**
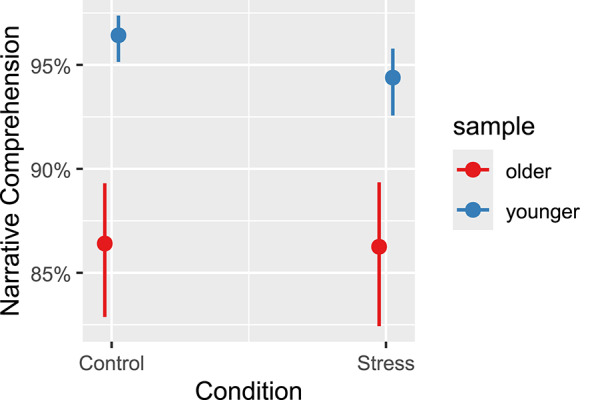
Predicted probabilities in the interaction model of condition and age.

We conducted a cross-experimental analysis comparing the two experiments to test for the age differences in narrative comprehension and the interaction effect of age (younger vs. older sample) and condition (stress vs. control). For this, we fitted for the binomial narrative comprehension variable generalized linear mixed-effects models (*glmer*) of the logit-family (*lme4*-package)^58^ for logistic regression, with condition (stress, control) and age group (younger vs. older sample) as fixed effects (main effect and interaction), and participants and item as random effects. We submitted the resulting model to a type 2 Anova using the Anova function of the car package^64^. Besides a significant main effect of condition (lower performance in stress condition, χ²(1) =  4.20, *p* =.040), and a significant effect of age group (lower performance for the older participants, χ²(1) =  130.08, *p* < .001), we observed a significant interaction effect of condition (stress, control), and age (younger, older), χ²(1) =  4.74, *p =* .029 (see Table 4). Whereas narrative comprehension was not influenced by acute stress in the older sample, we found a substantial impairment in the younger sample (see Fig. 8). This cross-experimental analysis confirms the results of Study 1 and 2.

**Table 4:** Summary of the generalized linear mixed model for the effects of age group (older vs. younger adults) x condition (control vs. stress).

*Predictors*	*Odds Ratios*	*SE*	β	*CI*	*z*	*p*
(Intercept)	6.74	0.95	6.74	5.11 – 8.90	13.49	< 0.001
condition [stress]	0.97	0.12	0.97	0.76 – 1.24	-0.23	0.818
age group [young]	3.95	0.57	3.95	2.98 – 5.23	9.60	< 0.001
condition [stress] * age group [young]	0.65	0.13	0.65	0.43 – 0.96	-2.18	0.029

Random Effect		
σ^2^	3.29	
τ*00 ID*	0.48	
τ*00 Story*	0.31	
ICC	0.19	
N *ID*	415	
N *Story*	24	
Observations	9960	
Marginal R^2^ / Conditional R^2^	0.081 / 0.258	

#### Difficulty level

**Fig. 9:**
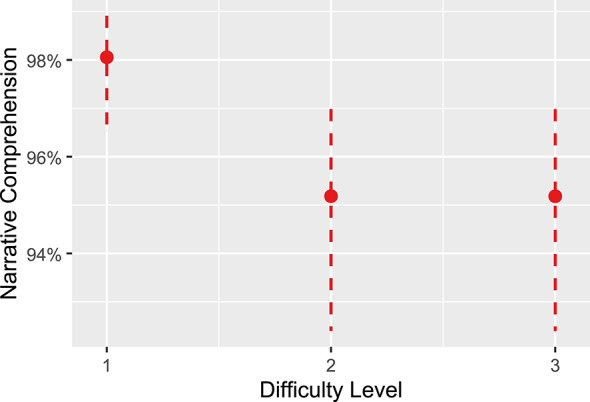
Average narrative comprehension performance (predicted probabilities for correct inferences) depending on the difficulty level (younger sample).

An exploratory analysis was conducted, exploring the hypothesis, that narrative comprehension decreases with a higher degree of difficulty. Firstly, a One-Way ANOVA was conducted in the younger group to compare mean differences between difficulty levels. The variance analysis showed that the level’s effect was significant with *F*(2, 404) =  7.36, *p* <.001. In order to further explore the significant ANOVA result, we decided to implement a GLMM to describe the data. Starting from an intercept-only model, which predicted narrative comprehension based solely on the overall mean across all participants, we sequentially added the fixed effect of difficulty level to investigate its influence on narrative comprehension. The comparison of both models showed a significant difference between the models: *X*^2^(2) =  16.13 *p* <.001, thus the difficulty level seems to be important for the narrative comprehension performance. Table 5 depicts the odds ratios, with confidence intervals and p-values of the extended model. Here we see that more difficult levels 2 and 3 result in poorer performance than level 1 (baseline category). Figure 9 demonstrates this result. 

**Table 5:** Difficulty level and comprehension performance (younger sample).

	*Odds Ratios*	*CI*	*P*
(Intercept)	26.29	20.03 – 34.49	< 0.001
Difficulty level 2	0.57	0.42 – 0.78	< 0.001
Difficulty level 3	0.59	0.44 – 0.81	0.001

Random Effect		
σ^2^	3.29	
τ*00 *	0.40	
ICC	0.11	
N	203	
Observations	4872	
Marginal R^2^ / Conditional R^2^	0.017 / 0.125	

**Fig. 10:**
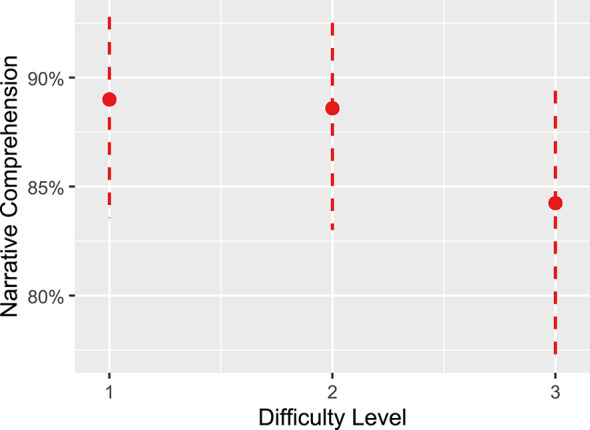
Average narrative comprehension performance (predicted probabilities for correct inferences) depending on the difficulty level (older sample).

Further, the same exploratory analysis was conducted in the older subsample, exploring the hypothesis, that narrative comprehension decreases with a higher degree of difficulty in both conditions. In order to test the hypothesis, we again implemented a GLMM. Starting from an intercept-only model, which predicted narrative comprehension based solely on the overall mean across all participants, we sequentially added the fixed effect of difficulty level to investigate its influence on narrative comprehension. The baseline model was compared with the extended model, including the difficulty level as a predictor. The comparison of both models showed no significant difference: *X*^2^(2) = 1.61, *p* =.448, thus the difficulty level does not seem to be important for the narrative comprehension performance. Table 6 depicts the odds ratios, with confidence intervals and p-values of the extended model. Figure 10 demonstrates this result.

**Table 6:** Difficulty level and comprehension performance (older sample).

*Predictors*	*Odds Ratios*	*CI*	*P*
(Intercept)	8.09	5.08–12.86	**< 0.001**
QUESTNNR [experimental]	0.88	0.62 – 1.25	0.484
Level [2]	0.96	0.51–1.80	0.899
Level [3]	0.66	0.36–1.23	0.191
QUESTNNR [experimental] ×Level [2]	1.03	0.70 – 1.53	0.871
QUESTNNR [experimental] ×Level [3]	1.26	0.86 – 1.84	0.239

**Random Effects**		
σ^2^	3.29	
τ*00 SERIAL*	0.54	
τ*00 Story*	0.33	
ICC	0.21	
N*SERIAL*	212	
N *Story*	24	
Observations	5088	
Marginal R^2^ / Conditional R^2^	0.006 / 0.215	

## Discussion

In this study, we examined narrative comprehension of pictorial stories in younger and older groups under stress and in a control condition. Consistent with H1, stress negatively affected comprehension in younger adults. In contrast, comprehension in older adults was not significantly modulated by stress, providing no support for H2. At the same time, however, we observed an overall age-related decline in narrative comprehension accuracy: although both groups performed at a high level, older adults scored around 86% correct, compared to 95% in younger adults. Thus, older adults were significantly less accurate overall. This pattern highlights a dissociation between general age-related decline in narrative comprehension accuracy and relative resilience to acute stress in older adults.

### Age-related decline in narrative comprehension

The overall reduction in comprehension accuracy observed in older adults is consistent with a broad literature documenting age-related declines in working memory, processing speed, and integrative processing. Such limitations likely contribute to difficulties in maintaining coherence across complex narratives, explaining why older adults performed worse overall. Our findings therefore align with accounts of normative cognitive aging that emphasize declining efficiency in fluid cognitive processes as a source of performance differences.

### Stress effects and age-related resilience

Despite this general decline, older adults’ narrative comprehension remained unaffected by stress. This finding mirrors previous work showing reduced stress sensitivity in older adults across cognitive domains. For example, Hidalgo and colleagues reported that stress impaired delayed free recall of pictures in younger but not older adults, suggesting age-related differences in vulnerability to stress-induced cognitive disruption^65^. Together, these findings indicate that aging may be associated with increased robustness to situational stressors, even in the presence of declining baseline performance.

This resilience is likely supported by compensatory mechanisms that offset age-related cognitive limitations. Older adults may rely more strongly on accumulated knowledge, well-established schemas, and richer event representations developed through life experience. Such resources can support comprehension by facilitating top-down processing and reducing reliance on effortful, resource-demanding integration processes. Evidence from reading research supports this idea, showing that older adults flexibly adapt their comprehension strategies to offset age-related declines, for example, by relying more strongly on contextual information and top-down predictions when processing becomes more demanding. Such language-general compensatory mechanisms may help explain why, in the present study, older adults’ narrative comprehension remained stable even under stress. Prior work demonstrates that older readers systematically adjust attentional allocation and inferential processing during comprehension, consistent with adaptive compensation rather than uniform decline^66–69^. This interpretation is also partially in line with prior work showing stability of narrative comprehension in aging^16,28,29^. Studies employing bridging inference tasks have found comprehension to remain relatively unaffected by age, even as other cognitive capacities show decline^16,29^. However, these studies were limited by their use of only a small set (eight stories) of child-oriented stories, restricting generalizability. Our findings extend this literature by demonstrating that when more demanding, age-appropriate, and thematically varied narrative materials are employed, age-related decline in comprehension becomes apparent, while resilience against stress remains evident.

### Integrating theoretical accounts

Theoretical accounts help explain this duality of decline and stability. The structure-building framework^1^ proposes that comprehension involves mapping incoming information onto existing mental representations; while aging may impair the efficiency of this process, older adults’ more differentiated schemas can provide compensatory support. Similarly, the SPECT framework^18^ emphasizes that current event models are shaped by stored event models in episodic memory; the sheer number and richness of such models in older adults may buffer against stress effects. From a psychometric perspective, this pattern reflects the distinction between fluid and crystallized intelligence^70–74^: although fluid abilities supporting rapid integration decline with age, crystallized knowledge and comprehension strategies remain stable or increase. Together, these accounts describe a shared mechanism whereby accumulated experience partially offsets declining processing efficiency, stabilizing comprehension under challenging conditions such as acute stress.

Another supporting perspective comes from the *default–executive coupling hypothesis of aging*^75^, which posits that aging involves a reorganization of cognitive networks characterized by increased interaction between executive control and default mode networks. This neural coupling supports the use of stored knowledge and semantic memory (crystallized intelligence) to guide cognitive performance when fluid abilities decline. In the context of visual narrative comprehension, the default–executive coupling hypothesis of aging helps explain how older adults may leverage prior experience and semantic schemas to maintain comprehension under acute stress, complementing the compensatory mechanisms described above.

### Emotional regulation and coping

Beyond cognitive compensation, emotional and motivational factors may also contribute to older adults’ resilience. According to *Socioemotional Selectivity Theory*^76^, aging is associated with a motivational shift toward emotionally meaningful goals and improved emotion regulation, which fosters adaptive emotion regulation and a focus on positive over negative information, a pattern known as the *positivity bias*. Older adults are therefore less likely to appraise experimental stressors as threatening and may experience reduced affective interference during cognitive tasks. Consistent with this view, older adults in the present study reported lower subjective stress levels than younger adults, and their confidence in responses remained stable under stress. Combined with greater life experience and coping resources, this could maintain performance under acute stress.

Coping research further supports this pattern. Coping capacities such as support-seeking, problem-solving, and distraction generally increase with age, and older adults often apply a wider repertoire of strategies compared to younger individuals^77,78^. Compensatory behaviors such as slowing down, increased concentration, and strategic resource allocation have been linked to higher levels of functioning in daily life and may help stabilize performance in demanding situations^79^. Compensation strategies are typically used proactively to postpone or reduce functional decline and may, in some cases, reflect an amplification of long-term habits^79^. By supporting everyday functioning, such strategies can also offset age-related declines in memory, processing speed, and executive functioning^80^, thereby stabilizing performance even under stressful conditions.

### Stress vulnerability in younger adults

Younger adults in our study showed greater vulnerability to stress, both subjectively and cognitively. At baseline, they reported significantly higher levels of perceived stress (PSS: *M* = 29.99 in younger adults vs. 23.05 in older adults) and subjective stress (*M* = 37.54 in the younger group vs. 12.37 in the older group). Stress also significantly reduced their comprehension accuracy and confidence in responses, whereas the comprehension accuracy and confidence levels of older adults remained stable. This finding highlights the broad negative impact of stress on younger adults, in line with prior research showing that stress correlates with reduced metacognitive accuracy, impairing individuals’ ability to assess their own performance^55^. Previous research also showed^81^, that stress results in reduced decision confidence when decisions involve moderate levels of uncertainty, but has no effect in cases of either high or low uncertainty. Large-scale surveys echo this pattern: for example, the APA’s 2023 Stress in America report showed that young adults (18–34) consistently report higher stress levels than older adults (65+)^82^. Likewise, Aldwin et al.^83^ found that middle-aged men were more likely to perceive problems as challenges or annoyances compared to older men.

### Biological considerations

Finally, biological differences in stress physiology may contribute to age-related differences in stress sensitivity. Aging is associated with changes in the hypothalamic-pituitary-adrenal (HPA) axis and related stress systems, leading to altered timing and magnitude of physiological responses. Specifically, older adults often show blunted or delayed cortisol reactivity compared to younger adults^84–86^. These age-related physiological changes may reduce the immediate impact of stress on cognitive performance, potentially contributing to the stability of narrative comprehension observed in older participants. While the current study did not directly measure physiological markers, existing evidence supports the notion that aging reshapes stress reactivity, complementing psychological and cognitive coping mechanisms in mitigating acute stress effects on cognition.

## Conclusion

Taken together, these findings suggest that younger adults may be more sensitive to the immediate cognitive costs of stress, whereas older adults, through a combination of adaptive cognitive and coping strategies and altered physiological reactivity, appear relatively protected. At the same time, from a broader perspective, older adults show lower narrative comprehension compared to younger adults.

## Limitations

One of the limitations of the current study is the lack of a broader variance. Especially for the sample of young people who were mainly university students, we can assume that they have above average cognitive skills (e.g., IQ, reading skills). Thus, they were probably more efficient in extracting relevant and suppressing irrelevant information^1^. In future studies, it would be interesting to manipulate education as one influencing factor^29^. Furthermore, the use of pictorial stimuli allows for the investigation of potential between-group differences, including among individuals with low literacy^87^.

Another limitation is the absence of measurement of a physiological stress marker. For example, salivary cortisol, as a marker of hypothalamus-pituitary-adrenal (HPA) activity, could be used to assess whether participants exhibit a physiological stress response^88^. By doing this, it would be possible to cover not only subjective but also objective stress assessment. Future studies would also benefit from collecting multiple stress measurements throughout the narrative comprehension task to ensure that the effects of stress induction persist for the entire duration of task engagement.

Another limitation is that the two age groups were tested in two separate experiments (including slightly different stress inductions). While this was a prerequisite of the local ethics committee, and the manipulation check still confirmed a sufficient increase in stress levels, the use of non-identical stress inductions may limit the comparability of group differences. Future research should aim to test different age groups within a single experimental framework using identical stress induction procedures, or ideally, examine a representative sample. Baseline stress levels also differed between age groups. However, the primary aim of the present study was to investigate the effects of acutely induced stress. Future research should further examine how baseline stress interacts with acute stress responses across the lifespan.

Moreover, the method used in this study explores only one aspect of narrative comprehension, namely bridging inference generation. While inferencing is crucial, it is not the sole process occurring during story comprehension. Therefore, future studies should employ different methods to measure narrative comprehension or explore alternative ways to test inference generation to support our findings. For example, open-ended questions or recall of the narrative instead of judging pre-determined inference would be one possible alternative to test narrative comprehension. By doing this, more insights into how inferences are generated and how visual narrative comprehension works in different groups could be gained.

The aim of this study was to investigate the process of narrative comprehension and the impact of stress on it in older and younger adults. In summary, our findings indicate that stress negatively affects both narrative comprehension and confidence in responses among younger adults, supporting Hypothesis 1 (H1). This result is consistent with cognitive load and stress-reactivity models, which predict that acute stress can impair the working memory and integrative processes required for narrative understanding.

In contrast, narrative comprehension and response confidence in older adults remained unaffected by stress, which does not support Hypothesis 2 (H2) but is in line with several theoretical perspectives. Socioemotional Selectivity Theory suggests that older adults’ motivational shift toward emotionally meaningful goals fosters adaptive emotion regulation and a reduced perception of threat, buffering against stress-related cognitive disruption. The default–executive coupling hypothesis of aging proposes that strengthened connectivity between executive control and default networks supports semantic and memory-based processing, which may help maintain comprehension under stress. Similarly, the structure-building framework and SPECT theory highlight the role of accumulated and differentiated event schemas, which older adults may possess in greater number, enabling efficient integration of new narrative information. Finally, the psychometric theory of fluid and crystallized intelligence, along with research on compensatory strategies, suggests that declines in fluid abilities may be offset by stable or enhanced crystallized knowledge and adaptive coping mechanisms, thereby preserving performance under challenging or stressful conditions^89–91^.

Taken together, these findings suggest that the effects of stress on narrative comprehension differ across the lifespan, with younger adults showing greater vulnerability to stress-induced impairments in both comprehension and confidence compared to older adults. These results have implications for designing age-tailored interventions to support narrative comprehension under high-stress conditions across all age groups.

## Data Availability

Data and syntaxes used to conduct analyses in a statistical package R are openly available at the project’s Open Science Framework page ([https://osf.io/5q3xc/]).

## References

[1] Gernsbacher, M. A., Varner, K. R. & Faust, M. E. Investigating differences in general comprehension skill. *J. Exp. Psychol. Learn. Mem. Cogn.* **16**, 430–445 (1990). 2140402 10.1037//0278-7393.16.3.430 PMC4301443

[2] Cohn, N. Visual narrative structure. *Cogn. Sci.* **37**, 413–452 (2013). 23163777 10.1111/cogs.12016

[3] Cohn, N. You’re a good structure, Charlie Brown: The distribution of narrative categories in comic strips. *Cogn. Sci.* **38**, 1317–1359 (2014). 24646175 10.1111/cogs.12116 PMC4159446

[4] Sandi, C. Stress and cognition. *WIREs Cogn. Sci.* **4**, 245–261 (2013). 10.1002/wcs.1222 26304203

[5] Salthouse, T. *Major Issues in Cognitive Aging* (Oxford University Press, 2009). 10.1093/acprof:oso/9780195372151.001.0001.

[6] Carstensen, L. L. The influence of a sense of time on human development. *Science* **312**, 1913–1915 (2006). 16809530 10.1126/science.1127488 PMC2790864

[7] Quintero Johnson, J. M. & Sangalang, A. Testing the explanatory power of two measures of narrative involvement: An investigation of the influence of transportation and narrative engagement on the process of narrative persuasion. *Media Psychol.* **20**, 144–173 (2017).

[8] Lynch, J. S. et al. The development of narrative comprehension and its relation to other early reading skills. *Read. Psychol.* **29**, 327–365 (2008).

[9] Bilandzic, H., Sukalla, F., Schnell, C., Hastall, M. R. & Busselle, R. W. The Narrative Engageability Scale: A Multidimensional Trait Measure for the Propensity to Become Engaged in a Story. *Int. J. Commun.* 32 (2019).

[10] Chang, S. & Egeth, H. E. Enhancement and suppression flexibly guide attention. *Psychol. Sci.* **30**, 1724–1732 (2019). 31693453 10.1177/0956797619878813

[11] Bower, G. H. & Morrow, D. G. Mental models in narrative comprehension. *Science* **247**, 44–48 (1990). 2403694 10.1126/science.2403694

[12] Kintsch, W. The role of knowledge in discourse comprehension: A construction-integration model. *Psychol. Rev.* **95**, 163–182 (1988). 3375398 10.1037/0033-295x.95.2.163

[13] Magliano, J. P., Larson, A. M., Higgs, K. & Loschky, L. C. The relative roles of visuospatial and linguistic working memory systems in generating inferences during visual narrative comprehension. *Mem. Cognit.* **44**, 207–219 (2016). 26450589 10.3758/s13421-015-0558-7

[14] Cohn, N. Being explicit about the implicit: Inference generating techniques in visual narrative. *Lang. Cogn.* **11**, 66–97 (2019).

[15] Hutson, J. P., Magliano, J. P. & Loschky, L. C. Understanding moment-to‐moment processing of visual narratives. *Cogn. Sci.* **42**, 2999–3033 (2018). 30447018 10.1111/cogs.12699 PMC6587724

[16] Varkentin, E., Brich, I.R., Sünkel, U. et al. Inference generation in older adults: Comparing pictorial and textual comprehension in the context of cognitive decline. *Mem Cogn* **54**, 27–44 https://doi.org/10.3758/s13421-025-01736-7 (2026). 10.3758/s13421-025-01736-7 PMC12864196 40681934

[17] Gernsbacher, M. A. & Faust, M. E. The mechanism of suppression: A component of general comprehension skill. *J. Exp. Psychol. Learn. Mem. Cogn.* **17**, 245–262 (1991). 1827830 10.1037//0278-7393.17.2.245 PMC4311900

[18] Loschky, L. C., Larson, A. M., Smith, T. J. & Magliano, J. P. The scene perception & event comprehension theory (SPECT) applied to visual narratives. *Top. Cogn. Sci.* **12**, 311–351 (2020). 31486277 10.1111/tops.12455 PMC9328418

[19] Magliano, J. P., Loschky, L. C., Clinton, J. & Larson, A. M. Is Reading the Same as Viewing? An Exploration of the Similarities and Differences Between Processing Text-and Visually Based Narratives. In *Unraveling the Behavioral, Neurobiological, & Genetic Components of Reading Comprehension* (Brookes Publishing Co, 2013).

[20] Cohn, N. & Kutas, M. What’s your neural function, visual narrative conjunction? Grammar, meaning, and fluency in sequential image processing. *Cogn. Res. Princ. Implic.* **2**, 27 (2017). 28603773 10.1186/s41235-017-0064-5 PMC5442195

[21] Schank, R. C. & Abelson, R. P. *Scripts, Plans, Goals, and Understanding* (Psychology Press, 2013). 10.4324/9780203781036.

[22] Cohn, N. Explaining ‘I can’t draw’: Parallels between the structure and development of language and drawing. *Hum. Dev.* **55**, 167–192 (2012).

[23] Cohn, N. & Structure Meaning, and Constituency in Visual Narrative Comprehension. (2012).

[24] Smith, M. E., Loschky, L. C. & Bailey, H. R. Eye movements and event segmentation: Eye movements reveal age-related differences in event model updating. *Psychol. Aging* **39**, 180–187 (2024). 37650795 10.1037/pag0000773 PMC10902178

[25] Noh, S. R. & Stine-Morrow, E. A. L. Age differences in tracking characters during narrative comprehension. *Memory & Cognition* **37**, 769–778 (2009). 19679857 10.3758/MC.37.6.769

[26] De Beni, R., Palladino, P., Borella, E. & Lo Presti, S. Reading comprehension and aging: Does an age-related difference necessarily mean impairment?. *Aging Clin. Exp. Res.* **15**, 67–76 (2003). 12841421 10.1007/BF03324482

[27] Van Boxtel, W. S. & Lawyer, L. A. Syntactic comprehension priming and lexical boost effects in older adults. *Lang. Cogn. Neurosci.* **38**, 105–120 (2023).

[28] Ulatowska, H. K., Chapman, S. B., Highley, A. P. & Prince, J. Discourse in healthy old-elderly adults: A longitudinal study. *Aphasiology* **12**, 619–633 (1998).

[29] Huff, M., Gagarina, N., Varkentin, E. & Brich, I. R. Education, not age, linked to narrative comprehension. *Learn. Instr.* **97**, 102102 (2025).

[30] Stefaniak, A. R., Blaxton, J. M. & Bergeman, C. S. Age differences in types and perceptions of daily stress. *Int. J. Aging Hum. Dev.* **94**, 215–233 (2022). 33739147 10.1177/00914150211001588

[31] Vasunilashorn, S., Lynch, S. M., Glei, D. A., Weinstein, M. & Goldman, N. Exposure to stressors and trajectories of perceived stress among older adults. *J. Gerontol. B. Psychol. Sci. Soc. Sci.* **70**, 329–337 (2015). 24906395 10.1093/geronb/gbu065 PMC4415078

[32] Hedgeman, E., Hasson, R. E., Karvonen-Gutierrez, C. A., Herman, W. H. & Harlow, S. D. Perceived stress across the midlife: Longitudinal changes among a diverse sample of women, the Study of Women’s health Across the Nation (SWAN). *Womens Midlife Health* **4**, 2 (2018). 29973982 10.1186/s40695-018-0032-3 PMC6027744

[33] Oakhill, J. V., Cain, K. & Bryant, P. E. The dissociation of word reading and text comprehension: Evidence from component skills. *Lang. Cogn. Process.* **18**, 443–468 (2003).

[34] McNamara, D. S. & Magliano, J. Chapter 9 Toward a Comprehensive Model of Comprehension. In *Psychology of Learning and Motivation* Vol. 51 297–384 (Elsevier, 2009).

[35] Brookshire, R. H. The discourse comprehension test. *Commun Ski Build. Psychol. Corp* (1993).

[36] Shields, G. S., Sazma, M. A. & Yonelinas, A. P. The effects of acute stress on core executive functions: A meta-analysis and comparison with cortisol. *Neurosci. Biobehav. Rev.* **68**, 651–668 (2016). 27371161 10.1016/j.neubiorev.2016.06.038 PMC5003767

[37] Oei, N. Y. L., Everaerd, W. T. A. M., Elzinga, B. M., Van Well, S. & Bermond, B. Psychosocial stress impairs working memory at high loads: An association with cortisol levels and memory retrieval. *Stress* **9**, 133–141 (2006). 17035163 10.1080/10253890600965773

[38] Xin, Z., Gu, S., Yi, L., Li, H. & Wang, F. Acute exposure to the cold pressor stress impairs working memory functions: An electrophysiological study. *Front. Psychiatry* **11**, 544540 (2020). 33329085 10.3389/fpsyt.2020.544540 PMC7719763

[39] Schoofs, D., Preuß, D. & Wolf, O. T. Psychosocial stress induces working memory impairments in an n-back paradigm. *Psychoneuroendocrinology* **33**, 643–653 (2008). 18359168 10.1016/j.psyneuen.2008.02.004

[40] Shields, G. S., Rivers, A. M., Ramey, M. M., Trainor, B. C. & Yonelinas, A. P. Mild acute stress improves response speed without impairing accuracy or interference control in two selective attention tasks: Implications for theories of stress and cognition.. *Psychoneuroendocrinology* **108**, 78–86 (2019). 31229636 10.1016/j.psyneuen.2019.06.001 PMC6707871

[41] Zanto, T. P. & Gazzaley, A. Neural suppression of irrelevant information underlies optimal working memory performance.. *J. Neurosci.* **29**, 3059–3066 (2009). 19279242 10.1523/JNEUROSCI.4621-08.2009 PMC2704557

[42] Oakhill, J., Yuill, N. & Parkin, A. On the nature of the difference between skilled and less-skilled comprehenders. *J. Res. Read.* **9**, 80–91 (1986).

[43] Dierolf, A. M., Fechtner, J., Böhnke, R., Wolf, O. T. & Naumann, E. Influence of acute stress on response inhibition in healthy men: An ERP study.. *Psychophysiology* **54**, 684–695 (2017). 28168718 10.1111/psyp.12826

[44] Shields, G. Does stress influence executive functions through mechanisms other than control itself? Examining the effects of stress on cognitive inhibition within the theory of emotional foundations of cognitive control. *Psychoneuroendocrinology* **153**, 106172 (2023).

[45] Jiang, C. & Rau, P.-L. The detrimental effect of acute stress on response inhibition when exposed to acute stress: An event-related potential analysis.. *Neuroreport* **28**, 922–928 (2017). 28777259 10.1097/WNR.0000000000000859

[46] Chang, J., Hu, J., Li, C. S. R. & Yu, R. Neural correlates of enhanced response inhibition in the aftermath of stress. *Neuroimage* **204**, 116212 (2020). 31546050 10.1016/j.neuroimage.2019.116212 PMC7509808

[47] Schwabe, L., Höffken, O., Tegenthoff, M. & Wolf, O. T. Stress-induced enhancement of response inhibition depends on mineralocorticoid receptor activation. *Psychoneuroendocrinology* **38**, 2319–2326 (2013). 23831264 10.1016/j.psyneuen.2013.05.001

[48] Yan, B. et al. EEG evidence of acute stress enhancing inhibition control by increasing attention. *Brain Sci.* **14**, 1013 (2024). 39452026 10.3390/brainsci14101013 PMC11505912

[49] Hasher, L. & Zacks, R. T. Working Memory, Comprehension, and Aging: A Review and a New View. In *Psychology of Learning and Motivation* Vol. 22 193–225 (Elsevier, 1988).

[50] Lustig, C., Hasher, L. & Zacks, R. T. Inhibitory deficit theory: Recent developments in a ‘new view’ In (eds Gorfein, D. S. & MacLeod, C. M.) (2007).

[51] Domes, G. & Zimmer, P. Acute stress enhances the sensitivity for facial emotions: A signal detection approach. *Stress* **22**, 455–460 (2019). 30938228 10.1080/10253890.2019.1593366

[52] Kirschbaum, C., Pirke, K.-M. & Hellhammer, D. H. The ‘Trier Social Stress Test’ – A tool for investigating psychobiological stress responses in a laboratory setting. *Neuropsychobiology* **28**, 76–81 (1993). 8255414 10.1159/000119004

[53] Degroote, C. et al. Acute stress improves concentration performance: Opposite effects of anxiety and cortisol. *Exp. Psychol.* **67**, 88–98 (2020). 32729405 10.1027/1618-3169/a000481

[54] Kan, Y. et al. Attentional blink affected by acute stress in women: The role of affective stimuli and attentional resources. *Conscious. Cogn.* **75**, 102796 (2019). 31374428 10.1016/j.concog.2019.102796

[55] Reyes, G., Silva, J. R., Jaramillo, K., Rehbein, L. & Sackur, J. Self-knowledge dim-out: Stress impairs metacognitive accuracy. *PLoS One* **10**, e0132320 (2015). 26252222 10.1371/journal.pone.0132320 PMC4529147

[56] Klever, L., Mamassian, P. & Billino, J. Age-related differences in visual confidence are driven by individual differences in cognitive control capacities. *Sci. Rep.* **12**, 6016 (2022). 35399123 10.1038/s41598-022-09939-7 PMC8995367

[57] Huff, M. et al. Cross-codal integration of bridging-event information in narrative understanding. *Mem. Cognit.* **48**, 942–956 (2020). 32342288 10.3758/s13421-020-01039-z PMC7381469

[58] Bates, D., Mächler, M., Bolker, B. & Walker, S. Fitting Linear Mixed-Effects Models Using lme4. *J Stat. Softw* **67**, (2015).

[59] Satow, L. SCI - Stress- und Coping-Inventar. Preprint at (2012). 10.23668/PSYCHARCHIVES.424

[60] Breyer, B. & Bluemke, M. Deutsche Version der Positive and Negative Affect Schedule PANAS (GESIS Panel). *Zusammenstellung Sozialwissenschaftlicher Items Skalen ZIS*. 10.6102/ZIS242 (2016).

[61] Cohen, S., Kamarck, T. & Mermelstein, R. A global measure of perceived stress. *J. Health Soc. Behav.* **24**, 385 (1983). 6668417

[62] Richer, R. et al. Stress+ – Towards an open-source web application for the remote induction of acute psychosocial stress. *Psychoneuroendocrinology* **160**, 106870 (2024).

[63] Clamor, A., Warmuth, A. M. & Lincoln, T. M. Arousal Predisposition as a Vulnerability Indicator for Psychosis: A General Population Online Stress Induction Study. *Schizophr. Res. Treat.* 1–8 (2015). (2015). 10.1155/2015/725136 PMC4493307 26199758

[64] Fox, J. & Weisberg, S. *An R Companion to Applied Regression* (Sage, 2010).

[65] Hidalgo, V. et al. Acute stress affects free recall and recognition of pictures differently depending on age and sex. *Behav. Brain Res.* **292**, 393–402 (2015). 26149415 10.1016/j.bbr.2015.07.011

[66] Rayner, K., Chace, K. H., Slattery, T. J. & Ashby, J. Eye movements as reflections of comprehension processes in reading. *Sci. Stud. Reading* **10**, 241–255 (2006).

[67] Rayner, K., Castelhano, M. S. & Yang, J. Eye movements and the perceptual span in older and younger readers. *Psychol. Aging* **24**, 755–760 (2009). 19739933 10.1037/a0014300

[68] Rayner, K., Yang, J., Schuett, S. & Slattery, T. J. Eye movements of older and younger readers when reading unspaced text. *Exp. Psychol.* **60**, 354–361 (2013). 23681016 10.1027/1618-3169/a000207

[69] Wang, J. et al. Adult Age Differences in Eye Movements During Reading: The Evidence From Chinese. *J. Gerontol. B Psychol. Sci. Soc. Sci.* **gbw036**10.1093/geronb/gbw036 (2016). 10.1093/geronb/gbw036 PMC6019021 27032427

[70] Cattell, R. B. Theory of fluid and crystallized intelligence: A critical experiment. *J. Educ. Psychol.* **54**, 1–22 (1963). 10.1037/h0024654 6043849

[71] Cattell, R. B. *Abilities: Their Structure, Growth, and Action* (Houghton Mifflin, 1971).

[72] Cattell, R. B. *Intelligence: Its Structure, Growth and Action* (North-Holland., 1987).

[73] Schubert, A.-L., Hagemann, D., Löffler, C. & Frischkorn, G. T. Disentangling the effects of processing speed on the association between age differences and fluid intelligence. *J. Intell.* **8**, 1 (2019). 31881681 10.3390/jintelligence8010001 PMC7151009

[74] Cavanaugh, J. C. & Blanchard-Fields, F. *Adult Development and Aging (5th Ed.).* (Wadsworth Publishing/Thomson Learning., (2006).

[75] Spreng, R. N. & Turner, G. R. The shifting architecture of cognition and brain function in older adulthood. *Perspect. Psychol. Sci.* **14**, 523–542 (2019). 31013206 10.1177/1745691619827511

[76] Carstensen, L. L. & DeLiema, M. The positivity effect: A negativity bias in youth fades with age. *Curr. Opin. Behav. Sci.* **19**, 7–12 (2018). 30327789 10.1016/j.cobeha.2017.07.009 PMC6186441

[77] Diehl, M., Coyle, N. & Labouvie-Vief, G. Age and sex differences in strategies of coping and defense across the life span. *Psychol. Aging*. **11**, 127–139 (1996). 8726378 10.1037//0882-7974.11.1.127

[78] Zimmer-Gembeck, M. J. & Skinner, E. A. Review: The development of coping across childhood and adolescence: An integrative review and critique of research. *Int. J. Behav. Dev.* **35**, 1–17 (2011).

[79] Tomaszewski Farias, S. et al. Compensation strategies in older adults: Association with cognition and everyday function. *American Journal of Alzheimer’s Disease & Other Dementias®* **33**, 184–191 (2018). 10.1177/1533317517753361 PMC10852491 29357670

[80] Harada, C. N., Natelson Love, M. C. & Triebel, K. L. Normal cognitive aging. *Clin. Geriatr. Med.* **29**, 737–752 (2013). 24094294 10.1016/j.cger.2013.07.002 PMC4015335

[81] Heereman, J. & Walla, P. Stress, uncertainty and decision confidence. *Appl. Psychophysiol. Biofeedback* **36**, 273–279 (2011). 21818600 10.1007/s10484-011-9167-9

[82] American Psychological Association. Stress in America 2023: A nation recovering from collective trauma. (2023). https://www.apa.org/news/press/releases/stress/2023/collective-trauma-recovery

[83] Aldwin, C. M., Sutton, K. J., Chiara, G. & Spiro, A. Age differences in stress, coping, and appraisal: Findings from the Normative Aging Study. *J. Gerontol. B. Psychol. Sci. Soc. Sci.* **51B**, P179–P188 (1996). 10.1093/geronb/51b.4.p179 8673639

[84] Wilkinson, C. W., Peskind, E. R. & Raskind, M. A. Decreased hypothalamic-pituitary adrenal axis sensitivity to cortisol feedback inhibition in human aging. *Neuroendocrinology* **65**, 79–90 (1997). 9032777 10.1159/000127167

[85] Mikneviciute, G. et al. Adult age differences in the psychophysiological response to acute stress. *Psychoneuroendocrinology* **153**, 106111 (2023). 37075654 10.1016/j.psyneuen.2023.106111

[86] Kudielka, B. M., Buske-Kirschbaum, A., Hellhammer, D. H. & Kirschbaum, C. HPA axis responses to laboratory psychosocial stress in healthy elderly adults, younger adults, and children: Impact of age and gender. *Psychoneuroendocrinology* **29**, 83–98 (2004). 14575731 10.1016/s0306-4530(02)00146-4

[87] Buddeberg, K. & Grotlüschen, A. *LEO 2018 – Leben Mit Geringer Literalität* (wbv Media GmbH & Company KG., 2020).

[88] Noack, H., Nolte, L., Nieratschker, V., Habel, U. & Derntl, B. Imaging stress: An overview of stress induction methods in the MR scanner. *J. Neural Transm.* **126**, 1187–1202 (2019). 30631946 10.1007/s00702-018-01965-y

[89] Horn, J. L. & Cattell, R. B. Age differences in fluid and crystallized intelligence. *Acta Psychol. (Amst.)* **26**, 107–129 (1967). 6037305 10.1016/0001-6918(67)90011-x

[90] Baltes, P. B. *Successful Aging: Perspectives from the Behavioral Sciences* (Cambridge University Press, 1990).

[91] Reuter-Lorenz, P. A. & Park, D. C. How does it STAC up? Revisiting the Scaffolding Theory of Aging and Cognition. *Neuropsychol. Rev.* **24**, 355–370 (2014). 25143069 10.1007/s11065-014-9270-9 PMC4150993

